# Association of sex-specific differences in lipoprotein(a) concentrations with cardiovascular mortality in individuals with type 2 diabetes mellitus

**DOI:** 10.1186/s12933-021-01363-x

**Published:** 2021-08-18

**Authors:** Marcello Ricardo Paulista Markus, Till Ittermann, Sabine Schipf, Martin Bahls, Matthias Nauck, Henry Völzke, Raul Dias Santos, Annette Peters, Tanja Zeller, Stephan Burkhard Felix, Ramachandran S. Vasan, Barbara Thorand, Elisabeth Steinhagen-Thiessen, Marcus Dörr

**Affiliations:** 1 Department of Internal Medicine B, University Medicine Greifswald, Greifswald, Germany; 2 German Centre for Cardiovascular Research (DZHK), Partner Site Greifswald, Greifswald, Germany; 3 German Center for Diabetes Research (DZD), Partner Site Greifswald, Greifswald, Germany; 4 Department of Study of Health in Pomerania/Clinical-Epidemiological Research, Institute for Community Medicine, University Medicine Greifswald, Greifswald, Germany; 5 Institute for Laboratory Medicine and Clinical Chemistry, University Medicine Greifswald, Greifswald, Germany; 6 Lipid Clinic, Heart Institute (InCor), University of São Paulo Medical School, São Paulo, Brazil; 7 Institute of Epidemiology, Helmholtz Zentrum München (German Research Center for Environmental Health), Neuherberg, Germany; 8 German Center for Diabetes Research (DZD), Partner Site München-Neuherberg, Neuherberg, Germany; 9 German Centre for Cardiovascular Research (DZHK), Partner Site Munich Heart Alliance, Munich, Germany; 10 Department for General and Interventional Cardiology, University Heart Center Hamburg, Hamburg, Germany; 11 German Centre for Cardiovascular Research (DZHK), Partner Site Hamburg/Lübeck/Kiel, Hamburg, Germany; 12 Boston University and National Heart, Lung, and Blood Institute’s Framingham Heart Study, Framingham, USA; 13 Preventive Medicine and Cardiology Sections, Evans Department of Medicine, Boston University School of Medicine, Boston, USA; 14 Department of Epidemiology, Boston University School of Public Health, Boston, USA; 15 Lipid Clinic at the Interdisciplinary Metabolism Center, Charité – University Medicine Berlin, Berlin, Germany; 16 Department of Internal Medicine B, Cardiology, Angiology, Pneumology and Internal Intensive Care Medicine, University Medicine Greifswald, Ferdinand-Sauerbruch-Straße, 17475 Greifswald, Germany

**Keywords:** Cardiovascular mortality, Dyslipidemia, Lipoprotein(a), Sex-specific, Type 2 diabetes mellitus

## Abstract

**Background:**

Compared to individuals without type 2 diabetes mellitus, the relative increase in cardiovascular mortality is much higher in women than in men in individuals with type 2 diabetes mellitus.

**Methods:**

We evaluated data from 7443 individuals (3792 women, 50.9%), aged 20 to 81 years, from two independent population-based investigations, SHIP-0 and MONICA/KORA S3. We analyzed the longitudinal sex-specific associations of lipoprotein(a) with cardiovascular mortality in individuals with and without type 2 diabetes mellitus using Cox regression.

**Results:**

During a median follow-up of 20.5 years (136,802 person-years), 657 participants (404 men and 253 women) died of cardiovascular causes. Among individuals without type 2 diabetes mellitus, men had a significantly higher risk for cardiovascular mortality compared to women in unadjusted model and after adjustment. On the other hand, in participants with type 2 diabetes mellitus, the risk for cardiovascular mortality was not different between men and women in the unadjusted model and after adjustment for age, body mass index, low-density lipoprotein-cholesterol, fasting status and study sample (SHIP-0, MONICA/KORA S3). Further adjustment for lipoprotein(a) concentrations had no impact on the hazard ratio (HR) for cardiovascular mortality comparing men versus women in individuals without type 2 diabetes mellitus [HR: 1.94; 95% confidence interval (CI) 1.63 to 2.32; p < 0.001]. In individuals with type 2 diabetes mellitus, however, further adjustment for lipoprotein(a) led to an increased risk for cardiovascular mortality in men and a decreased risk in women resulting in a statistically significant difference between men and women (HR: 1.53; 95% CI 1.04 to 2.24; p = 0.029).

**Conclusions:**

Women are described to have a stronger relative increase in cardiovascular mortality than men when comparing individuals with and without type 2 diabetes mellitus. Higher lipoprotein(a) concentrations in women with type 2 diabetes mellitus than in men with type 2 diabetes mellitus might partially explain this finding.

## Background

The leading causes of death in patients with type 2 diabetes mellitus (T2DM) are cardiovascular diseases (CVD) with the risk of cardiovascular (CV) mortality being two-fold that of individuals without T2DM [1]. Men have a greater absolute CV mortality risk than women, in both individuals without and with T2DM. On the other hand, when individuals with T2DM are compared to individuals without T2DM, the relative increase in CV mortality is much higher in women than in men [2, 3]. Several meta-analyses [4–8] showed that women with T2DM have a higher relative increase in the risk for incident fatal and non-fatal coronary heart disease and stroke than men with T2DM when compared with individuals without T2DM. One possible reason for these observed sex-related mortality differences in T2DM might be a worsening of CV risk factor profile during the transition from a normoglycemic state to T2DM in women (compared to men) [9].

Lipoprotein(a) (Lp[a]), which is synthesized in the liver, is a low-density lipoprotein (LDL)-like particle where the apolipoprotein B-100 is covalently bound to an additional apolipoprotein, the apolipoprotein(a) [10]. Noteworthy, Lp(a) is known to have atherogenic effects and is considered to be more important for CVD mortality [11, 12] than LDL-cholesterol (LDL-C). Interestingly, while there is evidence [11–16] that higher levels of Lp(a) are associated with CV morbidity, including aortic-valve calcification and stenosis, and CV mortality, the influence of diabetes on Lp(a) levels is still under debate [17–27]. Although the blood concentrations of Lp(a) are considered to be genetically determined by variations in the Lp(a) gene [28], previous studies [17, 18, 20, 22, 23, 26, 27, 29] have reported an inverse association of Lp(a) with dysglycemic conditions including T2DM.

To the best of our knowledge, no previous study had evaluated sex differences on the associations of Lp(a) with CV mortality stratifying individuals by the presence or not of T2DM. The aim of this study was to investigate the effect of sex on the associations of Lp(a) with CV mortality in individuals with and without T2DM, in two large population-based samples established in German regions with differing prevalence and incidence of T2DM.

## Methods

### Study populations

The present study is based on data from two population-based investigations, the Study of Health in Pomerania (SHIP) and the World Health Organization (WHO) Multinational Monitoring of Trends and Determinants in Cardiovascular Diseases (MONICA)/ Cooperative Health Research in the Region of Augsburg (KORA) survey 3 (S3) study.

### SHIP-0 (Northeastern Germany)

The Study of Health in Pomerania (SHIP) is a population-based prospective cohort study conducted in the Northeast of Germany. The presented analysis is based on data from the baseline examination SHIP-0 realized between 1997 and 2001. The study design has previously been described in detail [30, 31]. In brief, a sample from the adult population aged 20 to 79 years was randomly drawn using a multistage procedure. The total population comprised 212,157 inhabitants. A total of 7008 participants were sampled with 292 persons of each sex in each of the 12 5-year age strata. The net sample (without migrated or deceased persons) comprised 6265 eligible participants. Selected persons received a maximum of three written invitations. In case of non-response, letters were followed by a phone call or by home visits if contact by phone was not possible. The final SHIP-0 population comprised 4308 participants (2192 women, 50.9%; corresponding to a final response of 68.8%).

### MONICA/KORA S3 (Southern Germany)

The World Health Organization (WHO) Multinational Monitoring of Trends and Determinants in Cardiovascular Diseases (MONICA)/ Cooperative Health Research in the Region of Augsburg (KORA) survey 3 (S3) cohort study is a population-based study conducted in the area of Augsburg, Southern Germany [32]. List of municipalities and population registers from the city of Augsburg and the less urban regions “Landkreis Augsburg” and “Landkreis Aichach-Friedberg” were used as sampling frames for the first and the second stage of two-stage sampling, respectively. The second stage of sampling was stratified by sex and 10-year age group. The S3 baseline examination (1994–1995) included 4856 participants aged 25–74 years (2451 women, 50.5%; response rate: 74.9%).

### Pooled sample

While SHIP-0 is conducted in Northeast Germany, which has the highest T2DM prevalence and incidence in Germany, MONICA/KORA S3 was established in South Germany, which has the lowest type 2 diabetes mellitus (T2DM) prevalence and incidence. Our pooled sample, from SHIP-0 and MONICA/KORA S3, comprised 9164 individuals (4643 women, 50.7%) aged 20 to 81 years. For the present analyses, we excluded 1721 participants with missing data in any of the exposures, outcomes, confounders or stratification variables. Accordingly, our final analytical sample consisted of 7443 individuals (3792 women, 50.9%), aged 20 to 81 years.

All study participants provided written informed consent. The study was approved by the ethics committee of the University of Greifswald and the Bavarian Chamber of Physicians and complies with the Declaration of Helsinki.

### Interview, medical and laboratory examination

In both studies, information on age, sex, socio-economic variables, smoking habits and medical history was collected by trained and certificated medical staff during a standardized interview.

In both studies, T2DM at baseline was defined as self-reported diagnosis of the condition and/or current self-reported use of any hypoglycemic medication.

All participants underwent an extensive standardized medical examination including collection of blood samples. Anthropometric measurements included height and weight based on recommendations of the WHO [33]. Weight and height were measured to the nearest 0.1 kg and 0.5 cm, respectively, using calibrated weighing scales and stadiometers with the participant wearing light clothing and without shoes. Body mass index (BMI) was calculated as weight (kg)/height^2^ (m^2^). Waist circumference (WC) was measured to the nearest 0.1 cm using an inelastic tape midway between the lower rib margin and the iliac crest in the horizontal plane, with the participant standing comfortably with weight distributed evenly on both feet [34]. Waist-to-hip and waist-to-height ratios were calculated as the waist circumference divided by hip and height, respectively, measured in centimeters. After a resting period of at least five minutes, systolic and diastolic blood pressures as well as heart rate were measured three times on the right arm of seated participants using an oscillometric digital blood pressure monitor (HEM-705CP, Omron Corporation, Tokyo, Japan) in SHIP-0 and a random-zero sphygmomanometer [35] (Hawksley & Sons Ltd, Lancing, England) in MONICA/KORA S3 with an interval of three minutes between readings. Means of the second and third measurements for the systolic and diastolic blood pressures and for the heart rate were calculated and used for the present analyses. Hypertension was defined as systolic blood pressure ≥ 140 mm Hg and/or diastolic blood pressure ≥ 90 mm Hg and/or current self-reported use of any anti-hypertensive medication.

Venous blood samples were obtained from all study participants between 07:00 a.m. and 04:00 p.m. while sitting [36]. Time of the last meal was asked at the time of blood sampling, and the duration of fasting was calculated. Fasting status was defined as blood sampling after more or less/equal than 8 h since the last meal. Serum aliquots were stored at − 80 °C. All assays were performed according to the manufacturers’ recommendations by skilled technical personnel. In addition, the laboratory participates in official quarterly German external proficiency testing programs [37]. Lp(a) concentrations were measured by an immunoluminometric assay using two polyclonal antibodies directed against apolipoprotein(a) on a Magic Lite Analyzer II (Ciba Corning, Fernwald, Germany) [38] in SHIP-0 and using a fully automated, particle-enhanced turbidimetric immunoassay (Biokit Quantia Lp(a)—Test, Abbott Diagnostics, USA) in MONICA/KORA S3. Serum glucose levels were determined by a hexokinase method (GLUCO-quant®, Boehringer Mannheim, Mannheim, Germany with Hitachi 717 instrument in SHIP-0 and Hitachi 747 instrument in MONICA/KORA S3). Total cholesterol and supernatant cholesterol concentrations after a precipitation procedure using dextran sulphate (Immuno AG, Heidelberg, Germany) were measured by the CHOD-PAP method (Boehringer Mannheim, Mannheim, Germany with Hitachi 717 instrument in SHIP-0 and Hitachi 747 instrument in MONICA/KORA S3). Serum LDL-C levels were calculated as the difference of total cholesterol and supernatant cholesterol. Serum high-density lipoprotein-cholesterol levels were measured after the precipitation of the apolipoprotein B lipoprotein using magnesium chloride-phosphotungstic acid reagent by the CHOD-PAP method (Boehringer Mannheim, Mannheim, Germany with Hitachi 717 instrument in SHIP-0 and Hitachi 747 instrument in MONICA/KORA S3). Triglycerides levels were determined by an enzymatic color assay method (Boehringer Mannheim, Mannheim, Germany with Hitachi 717 instrument in SHIP-0 and Hitachi 747 instrument in MONICA/KORA S3).

Lipid-lowering medication was defined as current self-reported use of any lipid-lowering medication.

Smoking status was defined as current smokers (participants who, at the time of the interview, smoked at least 1 cigarette per day) and non-smokers [39].

### Information on vital status

Information on vital status was collected at regular intervals from time of enrolment into the study through March 31, 2019 in SHIP-0 and until 2016 in MONICA/KORA S3. Death certificates were requested from the local health authority at the place of death, and the underlying cause of death was independently coded according to the International Classification of Diseases version 10 (ICD-10). Participants were censored at death or loss to follow-up. The number of months between baseline examination and censoring was used as the duration of follow-up. The median duration of follow-up was 20.5 years (25th percentile: 18.9; 75th percentile: 21.1). During the 136,802 person-years of follow-up, 657 participants (404 men and 253 women) died of cardiovascular (CV) causes (ICD-10 codes: I10 to I79 and R99 in SHIP-0 and I10 to I99 in MONICA/KORA S3).

### Statistical analysis

Descriptive data was reported as median (25th and 75th percentile) for continuous variables and as absolute numbers and percentages for categorical variables stratified by T2DM and sex. Sex-specific associations of serum glucose levels with Lp(a) concentrations were analyzed using linear regression models adjusted for age, BMI, LDL-C, fasting status and study sample (SHIP-0, MONICA/KORA S3). In these analyses Lp(a) was log-transformed, because the residuals of the linear regression models were not normally distributed when using the untransformed Lp(a) variable.

Associations of sex with CV mortality were analyzed by Cox regression models in individuals with and without T2DM. We estimated three different models: the first unadjusted (adjusted only for study sample); the second additionally adjusted for age, BMI, serum LDL-C levels and fasting status and the third model further adjusted for Lp(a) concentrations. In individuals with T2DM the fit of the three models was compared using the Akaike Information Criterion (AIC), which penalizes the number of added variables to the model.

A two-sided p-value p < 0.05 was considered as statistically significant in all analyses. All calculations were performed using Stata 16.0 (Stata Corporation, College Station, TX, USA).

## Results

Our study sample comprised 455 (206 women, 45.3%) participants with T2DM and 6988 (3586, 51.3%) participants without T2DM (Table 1). In individuals without T2DM men were older, had a higher BMI, and higher levels of glucose, LDL-C and triglycerides and were more likely to have a history of hypertension, greater use of lipid-lowering medications and being current smokers. Noteworthy, while CV mortality was higher in men, Lp(a) levels were lower in men when compared with women. Among those with T2DM, men were more likely to be current smokers than women, while total cholesterol levels and use of antihypertensive medications were more frequent in women. Importantly, while there were no significant differences in CV mortality between men and women, Lp(a) concentrations were again lower in men when compared to women.

**Table 1:** Characteristics of the study sample stratified by type 2 diabetes mellitus status and sex (n = 7443)

Parameter	No type 2 diabetes mellitus (n = 6988)		Type 2 diabetes mellitus (n = 455)	
	Men	Women	Men	Women
	3402 (48.7%)	3586 (51.3%)	249 (54.7%)	206 (45.3%)
Age (years)	50 (37, 63)	47 (36, 60)	65 (56; 71)	66 (59; 72)
Cardiovascular mortality^a^ (%)	331 (9.7)	201 (5.6)	73 (29.3)	52 (25.2)
Lipoprotein (a) (mg/L)	80 (42; 216)	87 (48; 235)	70 (38; 204)	105 (51; 358)
Serum glucose (mmol/L)	5.3 (4.9, 5.8)	5.0 (4.7, 5.5)	8.6 (6.5; 11.8)	8.4 (6.1; 11.5)
Weight (kg)	83 (75, 91)	68 (60, 77)	85 (76; 95)	75 (66; 87)
Height (cm)	175 (170, 180)	162 (158, 167)	172 (167; 176)	159 (156; 163)
Body mass index (kg/m^2^)	27.0 (24.8, 29.5)	25.7 (22.7, 29.7)	29.1 (26.4; 31.6)	29.4 (26.3; 34.0)
Waist circumference (cm)	95 (88, 102)	81 (73, 91)	101 (95; 109)	94 (84; 102)
Waist-to-hip ratio	0.93 (0.88, 0.97)	0.79 (0.76, 0.84)	0.96 (0.92; 0.99)	0.86 (0.81; 0.89)
Waist-to-height ratio	0.54 (0.50, 0.59)	0.50 (0.44, 0.56)	0.59 (0.56; 0.63)	0.59 (0.53; 0.64)
Systolic blood pressure (mm Hg)	136 (125, 148)	125 (113, 140)	147 (135; 163)	145 (132; 156)
Diastolic blood pressure (mm Hg)	84 (76, 91)	79 (72, 86)	83 (75; 90)	81 (73; 87)
Hypertension (%)	1807 (53.2)	1299 (36.2)	205 (82.3)	166 (80.6)
Antihypertensive medications (%)	686 (20.1)	669 (18.7%)	133 (53.4)	131 (63.6)
Total cholesterol (mmol/L)	5.6 (4.9, 6.4)	5.6 (4.9, 6.4)	5.5 (4.8; 6.2)	5.8 (5.1; 6.7)
High-density lipoprotein cholesterol (mmol/L)	1.16 (0.96, 1.40)	1.47 (1.22, 1.76)	1.02 (0.82; 1.23)	1.13 (0.93; 1.39)
Low-density lipoprotein cholesterol (mmol/L)	3.5 (2.9, 4.2)	3.3 (2.6, 4.0)	3.4 (2.8; 4.0)	3.6 (2.8; 4.3)
Triglycerides (mmol/L)	1.82 (1.23, 2.73)	1.30 (0.93, 1.90)	2.40 (1.76; 3.45)	2.24 (1.56; 2.99)
Lipid-lowering medication (%)	181 (5.3)	135 (3.8)	34 (13.7)	38 (18.5)
Smoking (%)				
Non-smokers	2350 (69.1)	2702 (75.4)	205 (82.3)	185 (89.8)
Smokers	1050 (30.9)	883 (24.6)	44 (17.7)	21 (10.2)
Fasting > 8 h (%)	196 (5.8)	168 (4.7)	4 (1.6)	1 (0.5)

Data are reported as median, 25th, and 75th percentile for continuous data or as absolute numbers and percentages for categorical data

^a^Cardiovascular mortality = ICD-10 codes: I10 to I79 and R99 in SHIP-0 and I10 to I99 in MONICA/KORA S3

To test whether Lp(a) modifies the association of sex with CV mortality in individuals with T2DM, but not in those without, we introduced a three-term interaction of sex, Lp(a) and T2DM into a cox regression model which already included T2DM, LP(a), sex, age, BMI, LDL-C, fasting status and study sample. The p-value for this interaction was 0.090 which justified the stratification by T2DM status.

### Sex-specific associations of glucose values with Lp(a) concentrations

**Fig. 1:**
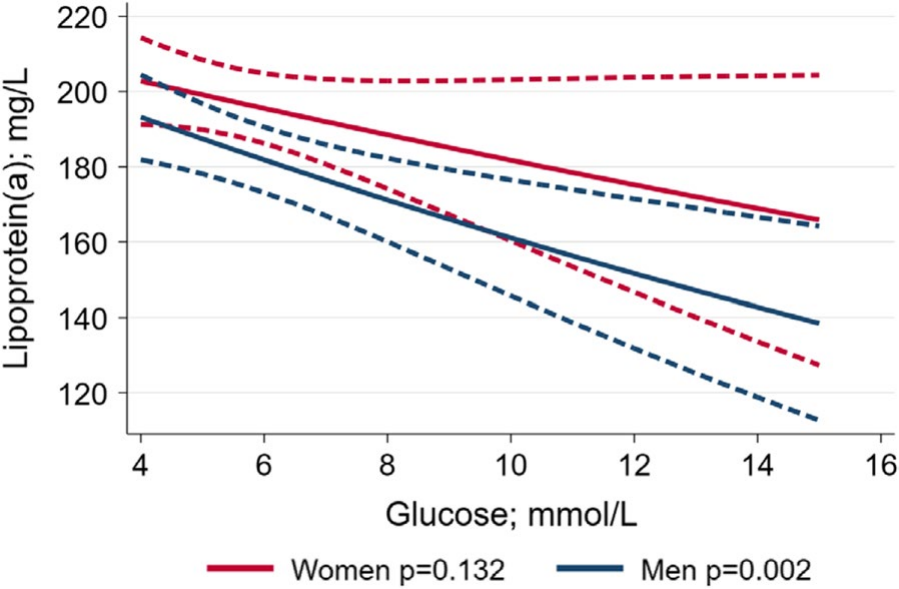
Adjusted* line (95% confidence interval) showing the sex-specific association between glucose levels and lipoprotein(a) concentrations (n = 7443). *Linear regression adjusted for age, BMI, LDL-C, fasting status and study sample

In multivariable linear regression models adjusted for age, BMI, LDL-C, fasting status and study sample; serum glucose levels were inversely associated with log-transformed Lp(a) levels in men [β = − 0.03; 95% confidence interval (CI) − 0.05 to − 0.01; p = 0.002] but not in women (β = − 0.02; 95% CI − 0.04 to 0.01; p = 0.132) (Fig. 1). In the total cohort, males have − 0.11 lower logarithmised Lp(a) than females in the fully adjusted model. The p-value for the interaction between serum glucose levels and sex on logarithmised Lp(a) was 0.018 indicating a significant interaction.

### Associations of sex with CV mortality stratified by T2DM status

**Fig. 2:**
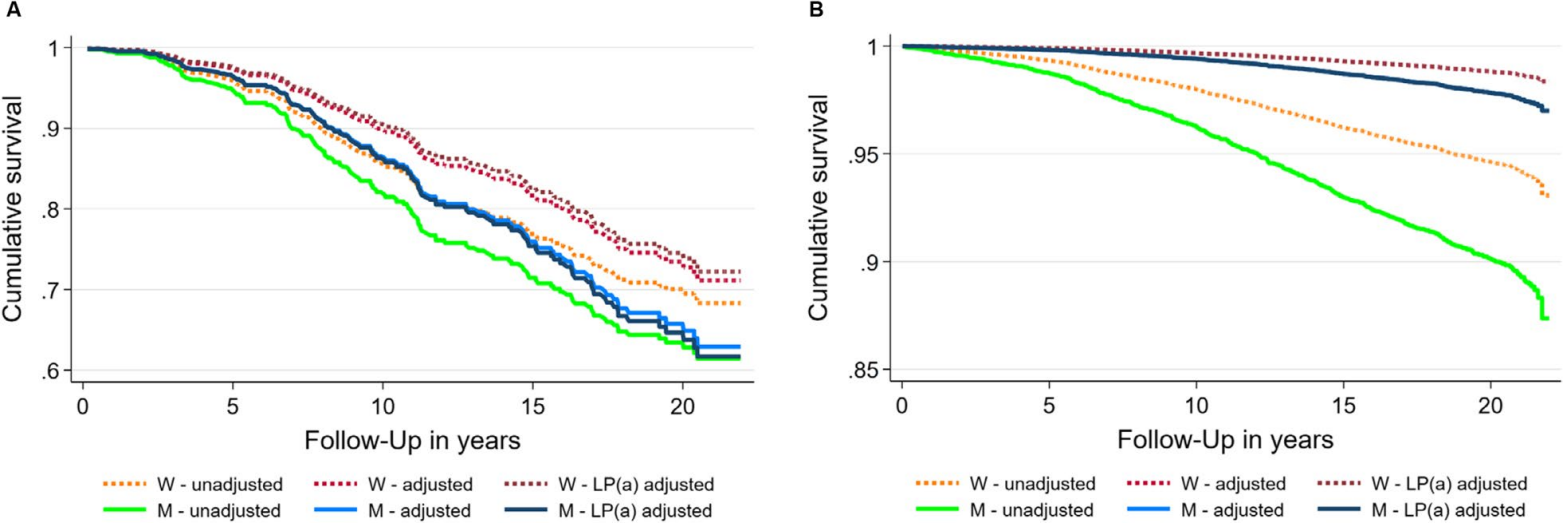
Unadjusted and adjusted* lines showing the association of sex with cardiovascular mortality^#^ in individuals with (**A**) and without (**B**) type 2 diabetes mellitus. *Cox linear regression unadjusted (adjusted only for study sample; model 1), adjusted for study sample, age, BMI, LDL-C and fasting status (model 2) and supplementary for Lp(a) levels (model 3). **There are overlap of the sex-specific lines for model 2 and 3 in B. ^#^Cardiovascular mortality = ICD-10 codes: I10 to I79 and R99 in SHIP-0 and I10 to I99 in MONICA/KORA S3. *M* men; *W* women

In participants without T2DM, the unadjusted (adjusted only for study sample [SHIP-0, MONICA/KORA S3]) Cox regression model 1 revealed a significantly higher CV mortality risk in men compared to women (Table 2). Moreover, the hazard ratio (HR) for the association of sex with CV death did not alter substantially after additional adjustment (model 2) for age, BMI, LDL-C, fasting status (model 2) plus Lp(a) levels (model 3) in individuals without T2DM (Table 2, Fig. 2B).

**Table 2:** Unadjusted and adjusted hazard ratio of the association of sex with cardiovascular mortality^a^ stratified by type 2 diabetes mellitus status (n = 7443)

	No type 2 diabetes mellitus (n = 6988)		Type 2 diabetes mellitus (n = 455)	
	Hazard ratio (95%-CI)	p-value	Hazard ratio (95%-CI)	p-value
Model 1				
Men versus women	1.88 (1.58; 2.24)	< 0.001	1.28 (0.89; 1.83)	0.178
Model 2				
Men versus women	1.94 (1.62; 2.31)	< 0.001	1.41 (0.97; 2.04)	0.070
Age (years)	1.15 (1.14; 1.16)	< 0.001	1.12 (1.09; 1.15)	< 0.001
Body mass index (kg/m^2^)	1.06 (1.04; 1.08)	< 0.001	1.00 (0.96; 1.04)	0.919
Low-density lipoprotein cholesterol (mmol/L)	1.05 (0.96; 1.14)	0.315	1.02 (0.85; 1.25)	0.773
Fasting status	1.28 (0.87; 1.88)	0.218	2.19 (0.68; 7.06)	0.191
Model 3				
Men versus women	1.94 (1.63; 2.32)	< 0.001	1.53 (1.04; 2.24)	0.029
Age (years)	1.15 (1.14; 1.16)	< 0.001	1.12 (1.09; 1.15)	< 0.001
Body mass index (kg/m^2^)	1.06 (1.04; 1.08)	< 0.001	1.00 (0.96; 1.04)	0.950
Low-density lipoprotein cholesterol (mmol/L)	1.04 (0.95; 1.14)	0.377	1.02 (0.84; 1.24)	0.840
Fasting status	1.27 (0.86; 1.88)	0.227	2.32 (0.72; 0.75)	0.161
Lipoprotein(a) (100 mg/L)	1.01 (0.98; 1.05)	0.553	1.07 (1.01; 1.12)	0.012

Data are reported as hazard ratios (95% confidence intervals [CI]) and p-values derived from Cox regression models. All models (including model 1) were also adjusted for study sample

^a^Cardiovascular mortality = ICD-10 codes: I10 to I79 in SHIP-0 and I10 to I99 in MONICA/KORA S3

On the other hand, in individuals with T2DM there was no statistically significant difference in CV mortality between men and women in the unadjusted (adjusted only for study sample [SHIP-0, MONICA/KORA S3]) model 1 (Table 2, Fig. 2A). Noteworthy, while the HR for CV mortality comparing men versus women changed from 1.28 (model 1) to 1.41 (model 2), after additional adjustment for age, BMI, LDL-C and fasting status; the difference between men and women was still not statistically significant. After further adjustment for Lp(a) levels, the sex-specific difference in risk for CV mortality became statistically significant and the HR increased from 1.41 to 1.53 (95% CI 1.04 to 2.24; p = 0.029) (Table 2, Fig. 2A). Specifically, in the T2DM group, Fig. 2A shows that while the full adjustment for the covariates (model 3), when compared with the unadjusted model 1, resulted in an increase in CV mortality in men, they lead to a decrease in women. The AIC’s values of the three models were 1425.1 (model 1), 1345.6 (model 2) and 1342.4 (model 3). The AIC decrease by 3.2 points from Model 2 to Model 3 can be interpreted as an increased fit of the model by including the LP(a) variable. A rule of thumb suggests that if the AIC is between 2 and 4 there is a considerable support for the more complicated model [40].

**Fig. 3:**
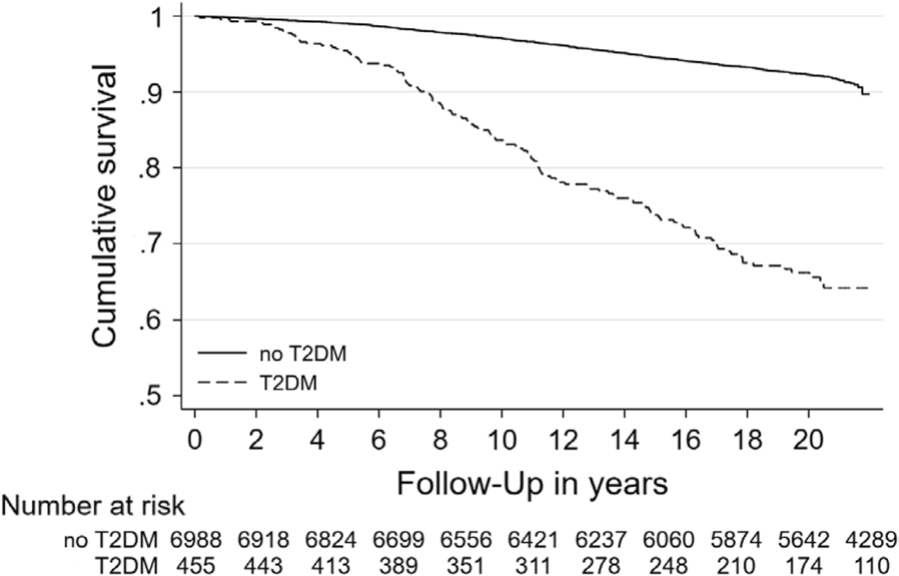
Kaplan–Meier curve for the total population stratified by type 2 diabetes mellitus

In sensitivity analyses (Table 3), we also further adjusted models 2 and 3 for hypertension and smoking status. While the HRs and the overall results did not alter significantly after these adjustments, we decided to not include hypertension and smoking status in our main analyses. The rationale behind our study was that women might have a stronger deterioration of CV risk factors through the evolution from a normoglycemic state to T2DM than men. Consequently, we included in our main models the risk factors that were more frequent in women than men which was not the case for hypertension and smoking. The adjustment for these two covariates would have led to an inappropriate (based in our rationale) increase in the risk for CV mortality in women with T2DM and a decrease in men with T2DM resulting in a lower HR for the association of sex with CV mortality. Also in sensitivity analyses (Fig. 3), we show the Kaplan-Meier curve for the total population stratified by type 2 diabetes mellitus.

**Table 3:** Unadjusted and adjusted hazard ratio of the association of sex with cardiovascular mortality^a^ stratified by type 2 diabetes mellitus status (n = 7443)

	No type 2 diabetes mellitus (n = 6988)		Type 2 diabetes mellitus (n = 455)	
	Hazard ratio (95%-CI)	p-value	Hazard ratio (95%-CI)	p-value
Model 1				
Men versus women	1.88 (1.58; 2.24)	< 0.001	1.28 (0.89; 1.83)	0.178
Model 2				
Men versus women	1.84 (1.54; 2.20)	< 0.001	1.36 (0.94; 1.97)	0.107
Age (years)	1.15 (1.14; 1.17)	< 0.001	1.12 (1.09; 1.15)	< 0.001
Body mass index (kg/m^2^)	1.06 (1.04; 1.08)	< 0.001	1.00 (0.96; 1.04)	0.934
Low-density lipoprotein cholesterol (mmol/L)	1.02 (0.94; 1.12)	0.599	1.05 (0.86; 1.28)	0.623
Fasting status	1.23 (0.83; 1.81)	0.306	1.50 (0.45; 4.98)	0.503
Hypertension	1.59 (1.28; 1.97)	< 0.001	1.59 (0.92; 2.76)	0.097
Smoking	2.10 (1.66; 2.65)	< 0.001	2.21 (1.31; 3.73)	0.003
Model 3				
Men versus women	1.84 (1.54; 2.20)	< 0.001	1.48 (1.01; 2.18)	0.034
Age (years)	1.15 (1.14; 1.17)	< 0.001	1.12 (1.09; 1.16)	< 0.001
Body mass index (kg/m^2^)	1.06 (1.04; 1.08)	< 0.001	1.01 (0.96; 1.05)	0.782
Low-density lipoprotein cholesterol (mmol/L)	1.02 (0.93; 1.11)	0.697	1.04 (0.85; 1.27)	0.691
Fasting status	1.22 (0.83; 1.80)	0.318	1.61 (0.49; 5.33)	0.433
Hypertension	1.59 (1.28; 1.98)	< 0.001	1.62 (0.94; 2.81)	0.083
Smoking	2.10 (1.66; 2.65)	< 0.001	2.28 (1.35; 3.84)	0.002
Lipoprotein(a) (100 mg/L)	1.01 (0.98; 1.05)	0.482	1.07 (1.02; 1.13)	0.006

Data are reported as Hazard Ratios (95% confidence intervals [CI]) and p-values derived from Cox regression models. All models (including model 1) were also adjusted for study sample

^a^Cardiovascular mortality = ICD-10 codes: I10 to I79 and R99 in SHIP-0 and I10 to I99 in MONICA/KORA S3

## Discussion

Our findings from two population-based studies indicate that sex differences in the association of Lp(a) levels with dysglycemia might, at least in part, explain the observation of various previous studies that the relative increase of CV mortality is stronger in women with T2DM than in men with T2DM when comparing individuals with and without T2DM, respectively. Specifically, we demonstrated a significant inverse association of glucose with Lp(a) concentrations in men, but no statistically significant association in women resulting in higher Lp(a) concentrations in women with T2DM than in men with T2DM (Fig. 1). Remarkably, in individuals with T2DM, adjustment for Lp(a) concentrations led to a significant decrease in the risk for CV mortality in women with T2DM and an increase in men with T2DM. These findings suggest that the higher relative increase in CV mortality observed in women with and without T2DM as compared to men with and without T2DM, might be due to the impact of higher Lp(a) concentrations in women than in men that have an effect in individuals with T2DM, but not in individuals without it.

To our knowledge, this is the first population-based study that analyzed sex-specific associations of glucose levels with Lp(a) concentrations and the effect of adjustment for Lp(a) on the estimated influence of sex on CV mortality in individuals with and without T2DM.

The associations of T2DM with sex-specific CV mortality are not full well-known. While male sex and T2DM are well established risk factors for higher CV morbidity and mortality, the impact of T2DM seems to be higher in women when compared to men [2, 3]. Our results are in line with this finding. A systematic review and meta-analysis [5] of 64 cohorts including 858,507 individuals and 28,203 coronary events showed that women with T2DM had 44% higher relative risk ratio for incident coronary heart disease than men with T2DM, when compared with individuals without T2DM.

Recently, a cohort study[2] consisting of all individuals of Denmark aged 40 to 89 years from Danish hospital databases, showed that when comparing participants with and without T2DM, women with T2DM had a 15% higher relative increase in risk for first event of myocardial infarction, heart failure, ischemic stroke and CV and all-cause mortality than men with T2DM.

Dyslipidemia is usually present in patients with T2DM through an association of cholesterol and triglycerides levels with atherosclerotic CVD [41]. Dyslipidemia in diabetic patients is usually characterized by increased concentrations of lipoproteins such as very low-density lipoprotein, chylomicrons, and small dense low-density lipoprotein cholesterol [42, 43]. On the other hand, the effects of T2DM on Lp(a) levels are still under discussion [17–27]. While the blood concentrations of Lp(a) seems to be genetically determined by variations in the Lp(a) gene [28], several studies [17–24] reported an inverse association of Lp(a) concentrations with prevalent or incident T2DM. While our analyses are in consonance with these results, we preferred to explore an inverse pathway for the associations, meaning that higher glucose concentrations were associated with lower Lp(a) levels. A large analysis [29] of 36 prospective studies with 126,634 participants found that women had 12% higher Lp(a) concentrations when compared with men, while patients with diabetes had 11% lower levels when compared with participants without diabetes. Unfortunately, sex-specific analyses regarding the relation between the levels of glucose and Lp(a) were not carried out.

We found just three studies [25–27] that analyzed sex-specific associations of dysglycemia with Lp(a) concentrations. Remarkably and absolutely in line with our results, a cross-sectional analysis of a Taiwanese population-based cohort study[26] with 1703 men and 1899 women aged 35 years and above showed a significant inverse association of fasting glucose and insulin (both fasting and 2-h post glucose intake) levels with Lp(a) in men, but not in women. Moreover, as in our study, women had higher Lp(a) concentrations than men. Another cross-sectional study [27] with a randomly-selected sample of 1167 men and women (mean age 61 years), showed a significant inverse association of insulin resistance (as assessed by the homeostasis model assessment-insulin resistance index [HOMA-IR]) with Lp(a) levels in men with diabetes, but not in women with diabetes. On the other hand, The Framingham Offspring Study [25] showed that while men had in absolute numbers a double inverse correlation of glucose levels with Lp(a) concentrations than women, it was not statistically significant. Importantly, this was an univariate analysis that did not consider the effects of other covariates like we did in our study.

The results of our analyses are in line with several previous studies [29, 44–48] which reported that increased blood levels of Lp(a) were independently associated with higher risk for CV mortality. A meta-analysis [49] with 56,804 individuals from seven prospective population-based cohorts from Europe with a follow-up to 24 years showed a HR of 1.49 (95% CI 1.29–1.73; p < 0.001) for first fatal or non-fatal major coronary event (acute myocardial infarction, coronary death, unstable angina pectoris and cardiac revascularization) and of 1.44 (95% CI 1.25–1.65, p < 0.001) for CVD (first fatal or non-fatal coronary heart disease event or likely cerebral infarction, coronary death, unstable angina pectoris, cardiac revascularization, ischaemic stroke, and unclassifiable death) when comparing Lp(a) levels ≥ 90th percentile with levels < 33rd percentile. A more recent analysis [48] with 69,761 individuals from two prospective studies of the Danish general population, the Copenhagen City Heart Study [44] and the Copenhagen General Population Study [19] showed that a 50 mg/dL (105 nmol/L) increase in Lp(a) concentrations was associated with a HR for CV mortality of 1.16 (95% CI 1.09 to 1.23).

At present we cannot explain with certainty the underlying mechanisms of our findings of an inverse association between glucose levels and Lp(a) concentrations. Of notice, this finding seems to be only significant in men, which may suggest that sex hormones might have an important effect.

A possible explanation would be that increased insulin levels, which are present in dysglycemic conditions, might decrease Lp(a) synthesis. An experimental study [50] with monkey hepatocyte cell cultures showed that an increase in insulin concentration resulted in a decrease in apo(a) synthesis by the hepatocytes through suppression of mRNA levels leading to lower Lp(a) concentrations [50]. Importantly, while this study [50] used simian hepatocytes from both male and female monkeys, the analyses were not sex-specific. Interestingly, a previous study [51] with the use of troglitazone, a hypoglycemic medication that reduces insulin levels, showed an increase in Lp(a) concentrations suggesting that insulin concentrations may have an effect on Lp(a) production. Again, there was no sex-specific analysis of these findings. Moreover, T1DM, which is characterized by insulin deficiency, is associated with higher levels of Lp(a) concentrations that return to normal levels after treatment with insulin [17, 50, 52]. On the other hand, we did not found any study that might explain a sex-specific difference in these results.

Another conceivable mechanism, which considers sex differences, is the size of bile acid pools. Women have a significant smaller bile acid pool than men [53]. This reduced bile acid pool might be even further decreased by hepatic insulin resistance, which will lead to a negative effect in the LXR–FXR (liver X receptor–farnesoid X receptor) axis activation, resulting in an increase in the apo(a) production [54].

A final thought might be linked to leptin levels. In our sample, the absolute differences in BMI, waist circumference, waist-to-hip ratio or waist-to-height ratio values were larger in women than men considering participants with or without T2DM. This means that leptin, which is produced by adipocytes, would have a higher increase in women than in men [55] during the transition from normoglycemia to T2DM and increased levels of leptin were associated with higher concentrations of Lp(a) [56]. Unfortunately, we did not have leptin measurements in our sample.

There are limitations of our study that require acknowledgment. First, the diagnosis of T2DM was partly self-reported, we cannot exclude possible misclassification even if we consider that pretty unlikely since we also considered the use of hypoglycemic medication in our definition of T2DM. Second, serial measurements of Lp(a) concentrations were not available, which could clarify the changes of the associations of glucose levels with Lp(a) concentrations over time and correct for potential regression dilution bias. However, it is important to recognize that Lp(a) levels are mostly genetically determined and, therefore, exhibit very limited intra-individual variability during life. Third, we used random glucose levels in our analyses. While we adjusted for fasting/non-fasting status, this correction might have not fully corrected the variability in blood glucose concentrations due to food intake. Fourth, we do not have a more specific measure of insulin resistance that could elucidate with more detail the associations observed. Fifth, our study is limited to white individuals of European ancestry; consequently, extrapolation to other ethnicities and age groups might be not suitable. Future longitudinal analyses and replication are warranted. Sixth, the diabetes group comprised only 455 pts (6% of the total population). Finally, even though we incorporated several confounders in our multivariable regression models, we cannot exclude unmeasured or unknown residual confounding.

Regardless of these limitations, our analyses have some important strengths, including the large number of individuals from two population-based studies with a wide age range, recruited from two regions with differing T2DM prevalence and incidence; and the availability of data on multiple metabolic risk factors that could be adjusted for.

## Conclusions

Women are described to have a stronger relative increase in CV mortality than men when comparing individuals with and without T2DM. Our findings from two large community-based studies suggest that this finding might be partially explained by our results of higher Lp(a) concentrations in women with T2DM than in men with T2DM.

## Data Availability

The datasets generated during and/or analysed during the current study are not publicly available due to data protection aspects but are available in an anonymized form from the corresponding author on reasonable request.

## Abbreviations

BMI: Body mass index
CI: Confidence interval
CV: Cardiovascular
CVD: Cardiovascular diseases
ICD-10: International Classification of Diseases version 10
LDL-C: Low-density lipoprotein cholesterol
Lp(a): Lipoprotein(a)
SHIP: Study of Health in Pomerania
T2DM: Type 2 diabetes mellitus
WC: Waist circumference
WHO: World Health Organization
WHO MONICA/KORA: World Health Organization Multinational Monitoring of Trends and Determinants in Cardiovascular Diseases/Cooperative Health Research in the Region of Augsburg survey study
